# Identification of immune biomarkers associated with basement membranes in idiopathic pulmonary fibrosis and their pan-cancer analysis

**DOI:** 10.3389/fgene.2023.1114601

**Published:** 2023-03-02

**Authors:** Chenkun Fu, Lina Chen, Yiju Cheng, Wenting Yang, Honglan Zhu, Xiao Wu, Banruo Cai

**Affiliations:** ^1^ Department of Respiratory and Critical Care Medicine, The Affiliated Hospital of Guizhou Medical University, Guiyang, China; ^2^ Guiyang Public Health Clinical Center, Guiyang, China; ^3^ Guizhou Medical University, Guiyang, China; ^4^ Department of Respiratory and Critical Care Medicine, The First People’s Hospital of Guiyang, Guiyang, China; ^5^ Shanghai Institute of Technology, Shanghai, China

**Keywords:** idiopathic pulmonary fibrosis, basement membrane, immune, pan-cancer, interstitial lung disease

## Abstract

Idiopathic pulmonary fibrosis (IPF) is a chronic progressive interstitial lung disease of unknown etiology, characterized by diffuse alveolitis and alveolar structural damage. Due to the short median survival time and poor prognosis of IPF, it is particularly urgent to find new IPF biomarkers. Previous studies have shown that basement membranes (BMs) are associated with the development of IPF and tumor metastasis. However, there is still a lack of research on BMs-related genes in IPF. Therefore, we investigated the expression level of BMs genes in IPF and control groups, and explored their potential as biomarkers for IPF diagnosis. In this study, the GSE32537 and GSE53845 datasets were used as training sets, while the GSE24206, GSE10667 and GSE101286 datasets were used as validation sets. In the training set, seven immune biomarkers related to BMs were selected by differential expression analysis, machine learning algorithm (LASSO, SVM-RFE, Randomforest) and ssGSEA analysis. Further ROC analysis confirmed that seven BMs-related genes played an important role in IPF. Finally, four immune-related Hub genes (*COL14A1*, *COL17A1*, *ITGA10*, *MMP7*) were screened out. Then we created a logistic regression model of immune-related hub genes (IHGs) and used a nomogram to predict IPF risk. The nomogram model was evaluated to have good reliability and validity, and ROC analysis showed that the AUC value of IHGs was 0.941 in the training set and 0.917 in the validation set. Pan-cancer analysis showed that IHGs were associated with prognosis, immune cell infiltration, TME, and drug sensitivity in 33 cancers, suggesting that IHGs may be potential targets for intervention in human diseases including IPF and cancer.

## 1 Introduction

Idiopathic pulmonary fibrosis (IPF) is a chronic progressive interstitial lung disease of unknown etiology (Richeldi et al., 2017). Its pathological features are diffuse alveolitis and alveolar structural damage, eventually forming honeycomb lung (Wolters et al., 2014). The clinical symptoms of IPF include dry cough, fatigue, and progressive exertional dyspnea. IPF is a rare disease, but its incidence is increasing and is more common in elderly male patients (Maher et al., 2021; Kondoh et al., 2022). Although disease progression varies greatly among individuals, the median survival after diagnosis is less than 3–5 years. Currently, treatment options for patients with IPF remain limited. Anti-fibrotic drugs (Pirfenidone and Nintedanib) have been approved to treat IPF, but they only slow the decline in lung function in IPF patients and do not improve survival (King et al., 2014; Richeldi et al., 2014). These drugs can also cause gastrointestinal adverse reactions, which limits their widespread use to some extent (Bargagli et al., 2019). At present, lung transplantation remains the only effective treatment. Unfortunately, not all patients are suitable for transplant, and complications following the transplant place a huge burden on patients.

Basement membranes (BMs) are a specialized form of extracellular matrix found in various organs of the human body, providing structural support for epithelium, endothelium, muscle, adipocytes, schwann cells, and axons (Mak and Mei, 2017). BMs are mainly composed of laminin and type IV collagen, which are linked together by nicolysaccharide and heparan sulfate proteoglycans to form different BMs in various tissues (Kruegel and Miosge, 2010). BMs can direct cell polarity, differentiation, migration and survival. For example, BMs can control epithelial growth and differentiation during embryonic development (Kyprianou et al., 2020). In cancer, the breakdown of the basement membrane promotes metastasis (Banerjee et al., 2022). Variations in the BMs gene are closely associated with many human diseases (Horton and Barrett, 2021; Wilson, 2022). BMs can mark the pathway of cell migration and epithelialization during tissue repair (Vracko, 1974; Rousselle et al., 2019). Changes in BMs homeostasis can lead to abnormal ECM aggregation and fibrosis (Wilson, 2020). Alveolar BMs promote gas exchange between alveoli and capillaries and regulate the function of cytokines and growth factors (West and Mathieu-Costello, 1999). The integrity of BMs maintains the normal lung structure and is critical for restoring alveolar epithelial homeostasis after lung injury (Strieter and Mehrad, 2009). However, a loss of alveolar and capillary BMs integrity was observed in IPF, suggesting that BMs are involved in IPF genesis (Chen et al., 2016).

Increasing evidence supports the important role of immune response in IPF. On the one hand, damage to lung epithelial cells leads to the production of pro-inflammatory cytokines such as IL-1 and IL-6 in M1 alveolar macrophages (Huang et al., 2018). These cytokines play an important role in host resistance to pathogen invasion. Inhibition of TNF-α secretion can alleviate bleomycin-induced pulmonary fibrosis and collagen deposition (Matsuhira et al., 2020). On the other hand, under chronic inflammatory conditions, Th2 cells secrete cytokines to gradually transform pro-inflammatory M1 macrophages into pro-fibrotic M2 macrophages (Shapouri-Moghaddam et al., 2018). M2 macrophages secrete multiple chemokines and activate Wnt/β-catenin signaling pathways leading to fibroblast activation, myofibroblast differentiation and extracellular matrix remodeling (Wynn and Vannella, 2016). Inhibition of Wnt/β-catenin signaling attenuates M2 macrophage-induced myofibroblast differentiation and bleomycin-induced pulmonary fibrosis (Hou et al., 2018). Another study found that vaccination inhibited M2 macrophage production and fibrocyte recruitment in bleomycin-induced pulmonary fibrosis (Collins et al., 2012). Moreover, the importance of immune responses in IPF has been confirmed by genetic studies, such as DEP domain containing MTOR interacting protein (*DEPTOR*) increase the risk or susceptibility of IPF(Allen et al., 2020).

**FIGURE 1:**
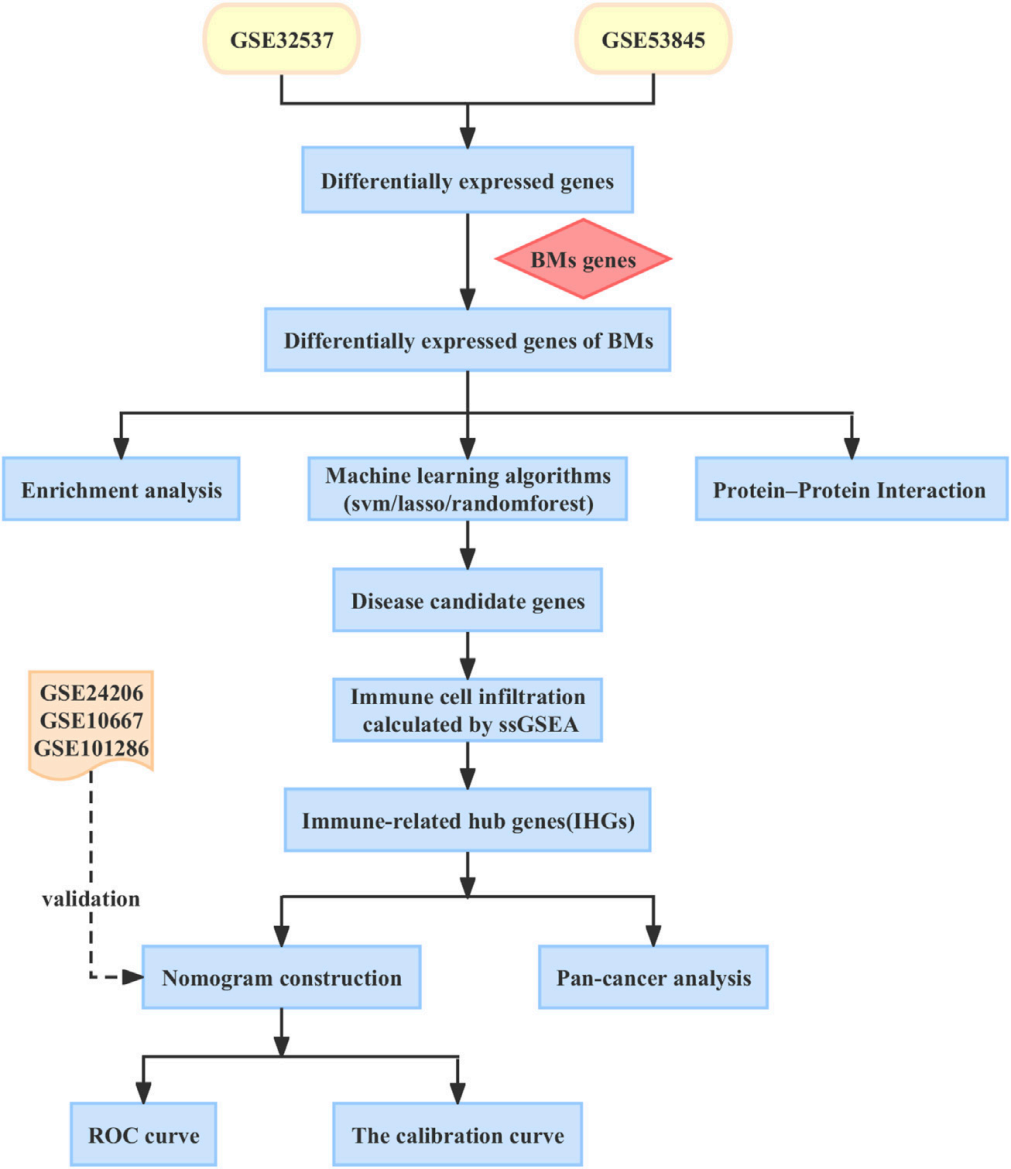
The workflow chart of our study.

In this article, we aim to explore the immune markers associated with BMs in IPF and construct a nomogram model to predict the risk of IPF in patients. Increased evidence suggests that IPF is closely linked to cancer (Ballester et al., 2019; Tzouvelekis et al., 2019). However, little is known about the relationship between IPF and cancer. Therefore, we conducted an in-depth analysis of the role of BMs-related immune biomarkers in pan-cancer to explore the common pathogenesis of IPF and cancer, and to find potential therapeutic targets for patients with IPF and cancer. In Figure 1, you can see the workflow chart.

## 2 Materials and methods

### 2.1 Data download and processing

We obtained 222 BMs related genes from previous studies (Jayadev et al., 2022). We obtained datasets numbered GSE32537, GSE53845, GSE24206, GSE10667 and GSE101286 from the GEO database (https://www.ncbi.nlm.nih.gov/geo/). The GSE32537, GSE53845 datasets were used as training set. Datasets GSE24206, GSE10667 and GSE101286 were used as validation sets for independent external validation. The main features of the five datasets were shown in Table 1. Datasets were merged after identity document transformation. The original data was normalized through R software “sva” package, followed by classification of the datasets into two categories: IPF and control groups. Finally, we evaluated the quality of the dataset using principal component analysis and plotted the PCA plot *via* R software “ggplot2” package.

**TABLE 1:** An overview of the main features of the dataset used in this study.

Dataset	Platform	IPF	Normal	Publication years	Used for
GSE32537	GPL6244	167	50	2013	DEGs analysis
GSE53845	GPL6480	40	8	2014	DEGs analysis
GSE24206	GPL570	17	6	2011	Model validation
GSE10667	GPL4133	31	15	2009	Model validation
GSE101286	GPL6947	12	3	2017	Model validation

### 2.2 Identification of differentially expressed genes in BMs

We screened differentially expressed genes (DEGs) through R software “limma” package, filter condition for *p* < 0.05 and | log_2_FC | > 1. A volcano map was drawn through the “ggplot2” package of R software to show DEGs. The “VennDiagram” package in R was used to obtain the intersection of BMs-related genes and DEGs, and the differentially expressed genes of basement membranes (BMDEGs) were obtained.

### 2.3 Protein–protein interaction (PPI) and enrichment analysis of BMDEGs

We visualized the PPI network of BMDEGs *via* the STRING database and performed GO, KEGG, and DO enrichment analyses of BMDEGs *via* the “ClusterProfiler” and “DOSE” packages of R software.

### 2.4 Three machine learning algorithms for screening disease candidate genes

We used LASSO, SVM-RFE and RandomForest machine learning algorithms to screen disease candidate genes. LASSO analysis was performed using 10-fold cross-validated penalty parameters *via* the “glmnet” package of R software. The minimal bimomial deviation was used to determine the optimal penalty parameter lambda. SVM-RFE algorithm detects the points with the minimum cross-validation error through the “e1071”, “kernlab” and “caret” packages in R software to screen disease candidate genes. The RandomForest algorithm uses the “randomforest” package of R software to screen disease candidate genes. The Venn diagram visualizes disease candidate genes obtained from the results of three machine learning algorithms.

### 2.5 Validation of disease candidate genes

To understand the specificity and sensitivity of disease candidate genes for IPF diagnosis, we draw the receiver operating characteristic (ROC) curve through the “pROC” package of R software. Results were presented in the form of area under the curve (AUC). If the AUC of candidate genes was greater than 0.6, we believed that it had diagnostic significance for IPF. Disease candidate gene expression in the IPF and control groups was shown in the box plot.

### 2.6 Analysis of immune infiltration

We performed correlation analysis of immunity through the “corrplot” package of R software and plotted the correlation heatmap. R software “ggpubr” and “reshape2” packages were used to analyze the differential expression of immune cells and immune functions in IPF and control groups. Spearman correlation analysis was conducted through the “psych” and “ggcorrplot” packages of the R software to analyze the correlation between disease candidate genes and immunity. The screening criteria for IPF immune-related hub genes (IHGs) were more than 1/2 immune infiltration and the correlation coefficient was greater than 0.2.

### 2.7 Establishment and validation of IHGs risk model

We constructed the nomogram of IHGs and plotted the calibration curve to determine the reliability of the nomogram through the “rms” package of R software. ROC curves were plotted to assess the accuracy of IHGs in diagnosing IPF *via* the “ROCR” package of R software.

### 2.8 Differential analysis of IHGs in human cancer

We downloaded tumor transcriptome data, clinical data, immune subtype data, mutation data and stemness score (RNAss and DNAss) from the UCSC Xena database (https://xenabrowser.net/). The expression levels of IHGs in 33 tumor and adjacent samples were extracted using the “limma” package of R software. Then, we retained tumor types with more than 5 paracancer tissues, and analyzed the expression of IHGs in tumors and paracancer tissues through the “ggpubr” package of R software.

### 2.9 Survival analysis of IHGs in pan-cancer

COX regression analysis was conducted through the “survival” package of R software to determine whether IHGs expression was correlated with the survival time and survival status of cancer patients, and the results were presented in forest plots. Survival analysis was performed through the “survival” and “surminer” packages of R software to determine whether IHGs expression was linked with the prognosis of tumor patients.

### 2.10 Mutation analysis of IHGs in pan-cancer

Tumor mutation burden (TMB) and microsatellite instability (MSI) is specific indicators for predicting immunotherapy in cancer patients. Therefore, we used the “fmsb” package of R software to plot radar maps of TMB and MSI to determine the correlation between IHGs expression and 33 types of tumors.

### 2.11 Tumor microenvironment (TME) and tumor stemness analysis

We calculated the immune score, stromal score, and estimated score for each sample in the tumor using the “estimate” package of R software. The correlation between IHGs expression and purity in 33 tumors was analyzed by Spearman correlation analysis. The relationship between IHGs expression and tumor stemness was determined by the “corrplot” package of R software.

### 2.12 Immune analysis of IHGs in pan-cancer

Immune subtypes including C1(Wound Healing), C2(IFN γ Dominant), C3(Inflammatory), C4 (lymphocyte Depleted), C5 (M2 macrophages Dominant), C6 (TGF-β Dominant) subtypes. Previous studies have shown that among the six immune subtypes, C4 and C6 are associated with lower survival rates, while C3 and C5 are the opposite (Tamborero et al., 2018). The relationship between IHGs expression and immune subtypes was analyzed by the “limma”, “ggplot2” and “reshape2” packages of R software. The correlation between IHGs expression and immune checkpoints was completed by Pearson correlation analysis. Finally, we investigated the correlation between IHGs expression in tumors and 21 types of immune cells using TIMER2.0 database (http://timer.cistrome.org/).

### 2.13 Enrichment analysis

We used the GeneMANIA database (http://genemania.org/) to predict and visualize genes that function similar to IHGs. The Metascape website (https://metascape.org/) was used to analyze the functions in which genes may be involved.

### 2.14 Drug sensitivity analysis of IHGs in pan-cancer

We downloaded drug sensitivity data for 60 human cancers from the CellMiner websites (https://discover.nci.nih.gov/cellminer/) and screened 263 FDA-approved or clinical trial drugs for this study. The relationship between IHGs expression and drugs was analyzed by Pearson correlation analysis.

### 2.15 Statistical analysis

Statistical tests were performed using R software (version 4.1.3). For all statistical analyses, *p < 0.05* was considered statistically significant (“***”, “**”, “*”, “ns” are “*p < 0.001*” “*p < 0.01*” “*p < 0.05*” “no significance”). Relevant scripts and supported data can be seen on the Github website (https://github.com/fuchenkun/Basement-membranes).

## 3 Results

### 3.1 Screening for BMDEGs of IPF

**FIGURE 2:**
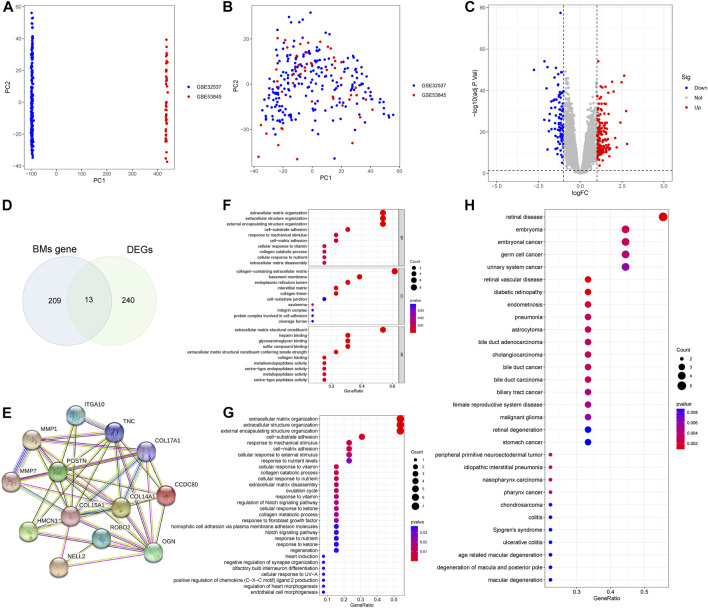
Differential expression analysis and enrichment analysis. **(A)** PCA plot shows training set before batch effect. **(B)** PCA plot shows the training set after batch effect. **(C)** A volcanic map of DEGs. **(D)** The Venn diagram of BMs related genes and DEGs. **(E)** Protein interaction network of BMDEGs. GO function**(F)**, KEGG pathway**(G)** and Disease enrichment analysis**(H)** of BMDEGs.

We combined GSE32537, GSE53845 datasets and corrected the batch effects for subsequent analyses (Figures 2A, B). 253 DEGs were screened, of which 158 genes were over-expressed and 95 genes were under-expressed. The results of the DEGs were presented as a volcano map (Figure 2C). A Venn diagram was also created, which showed that 13 BMDEGs, of which 12 were upregulated and 1 downregulated (Figure 2D).

### 3.2 Protein interaction network and enrichment analyses of BMDEGs

We constructed a protein interaction network for BMDEGs (Figure 2E). Then we performed enrichment analyses to better understand the functions, pathways, and diseases that BMDGEs might be involved in. As shown in Figure 2F, our results indicated that biological processes were mainly related to the structure of extracellular matrix, collagen catabolic processes, and responses to mechanical stimulus. The cellular components mainly involved extracellular matrix, basement membrane and endoplasmic reticulum. Extracellular matrix structural constituent, heparin binding, glycosaminoglycan binding, sulfur compound binding and extracellular matrix structural constituent conferring tensile strength were significantly enriched in molecular functions. The KEGG analysis revealed that BMDEGs tended to be enriched in the following terms: extracellular matrix organization, extracellular structure organization, external encapsulating structure organization, cell−substrate adhesion and response to mechanical stimulus (Figure 2G). Moreover, DO analysis found that BMDEGs were specifically enriched in IPF and were also associated with endocrine disorders, reproductive system diseases, and cancer (Figure 2H).

### 3.3 Screening and validation for IPF diagnostic markers

**FIGURE 3:**
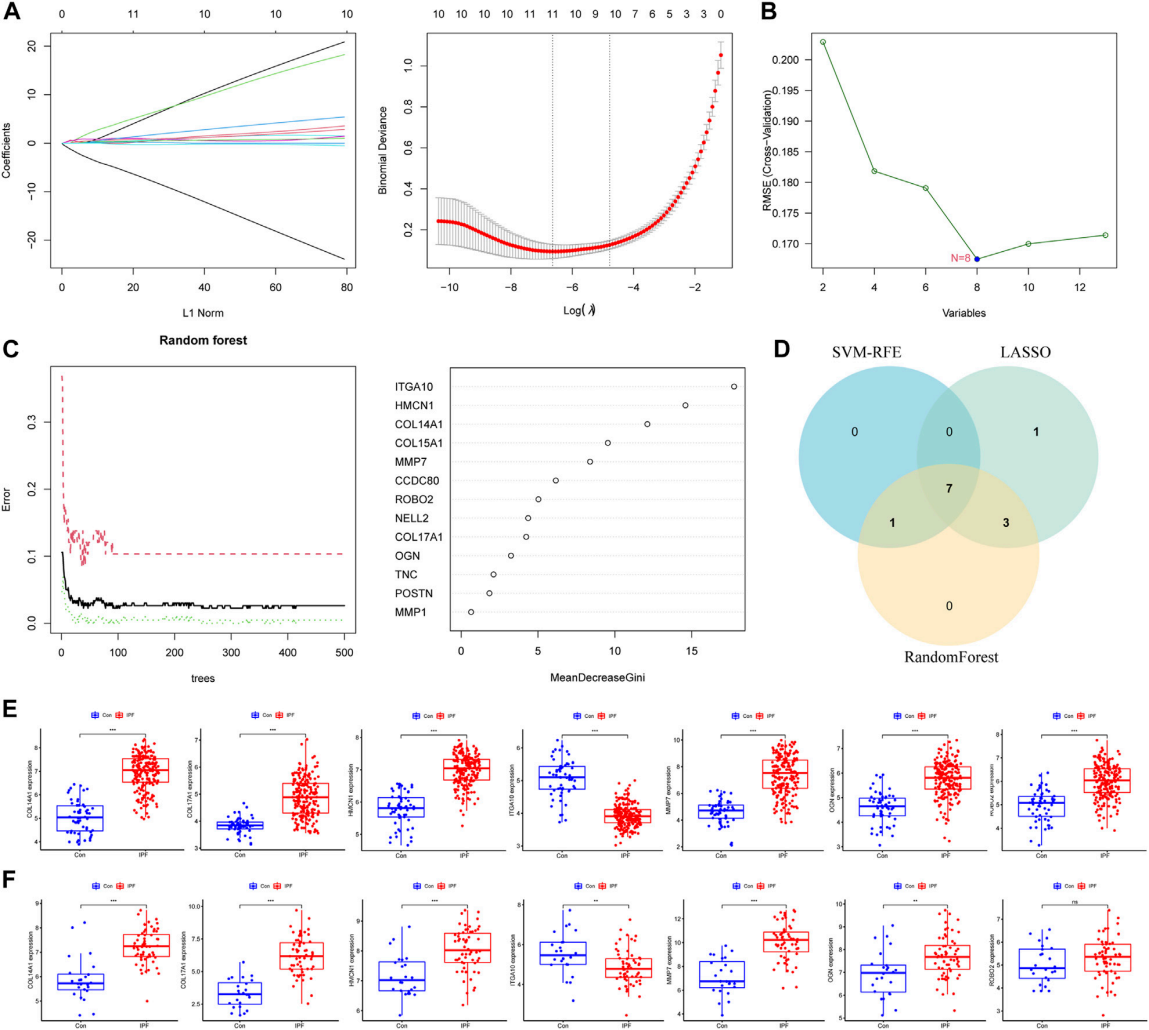
Machine learning algorithms identify disease candidate genes. **(A)** LASSO model for screening candidate genes for disease. **(B)** SVM-RFE algorithm for screening disease candidate genes. **(C)** Random forest model to screen candidate genes for disease. **(D)** The Venn diagram shows the common disease candidate genes of LASSO, RandomForest and SVM-RFE algorithms. **(E)** Boxplot representing expression of disease candidate genes in training set. **(F)** Boxplot representing expression of disease candidate genes in the validation set.

We further used machine learning algorithms to screen disease candidate genes from BMDEGs. LASSO regression analysis selected 11 genes (Figure 3A), the SVM-RFE algorithm identified eight genes (Figure 3B), and RandomForest screened 11 genes (Figure 3C). Finally, through the gene intersection obtained by the three algorithms, seven disease candidate genes (*COL14A1*, *COL17A1*, *HMCN1*, *ITGA10*, *MMP7*, *OGN* and *ROBO2*) were identified (Figure 3D). Then we analyzed the expression of seven disease candidate genes in the training group and validated them using external datasets. Validation group dataset eliminates batch effect for subsequent analysis (Supplementary Figure S1). As presented in Figure 3E, the boxplot showed that seven disease candidate genes were significantly upregulated in IPF groups and 1 candidate gene was significantly downregulated in IPF groups. We saw the same results in the validation dataset, but *ROBO2* was not statistically significant (Figure 3F). We further performed ROC analysis to examine the diagnostic efficacy of seven disease candidate genes for IPF. The results suggest that the seven disease candidate genes have diagnostic value in distinguishing IPF groups from control groups: *COL14A1* (AUC = 0.964), *COL17A1* (AUC = 0.915), *HMCN1* (AUC = 0.961), *ITGA10* (AUC = 0.946), *MMP7* (AUC = 0.937), *OGN* (AUC = 0.894) and *ROBO2*(AUC = 0.856) (Supplementary Table S1). Similarly, we evaluated the diagnostic efficacy of seven disease candidate genes for IPF in the validation group dataset using ROC analysis. The results indicated that AUCs of the disease candidate genes were *COL14A1* (AUC = 0.881), *COL17A1* (AUC = 0.949), *HMCN1*(AUC = 0.813), *ITGA10* (AUC = 0.707), *MMP7* (AUC = 0.910), *OGN* (AUC = 0.719) and *ROBO2* (AUC = 0.600) (Supplementary Table S2). In conclusion, the AUC values of *COL14A1*, *COL17A1*, *HMCN1*, *ITGA10*, *OGN* and *MMP7* in the training dataset and validation dataset were all greater than 0.7. These results suggest that the candidate genes are closely related to IPF and have the potential to be used as biomarkers of IPF and indicators to evaluate the efficacy of patients.

### 3.4 Immune infiltration analysis and IHGs screening

**FIGURE 4:**
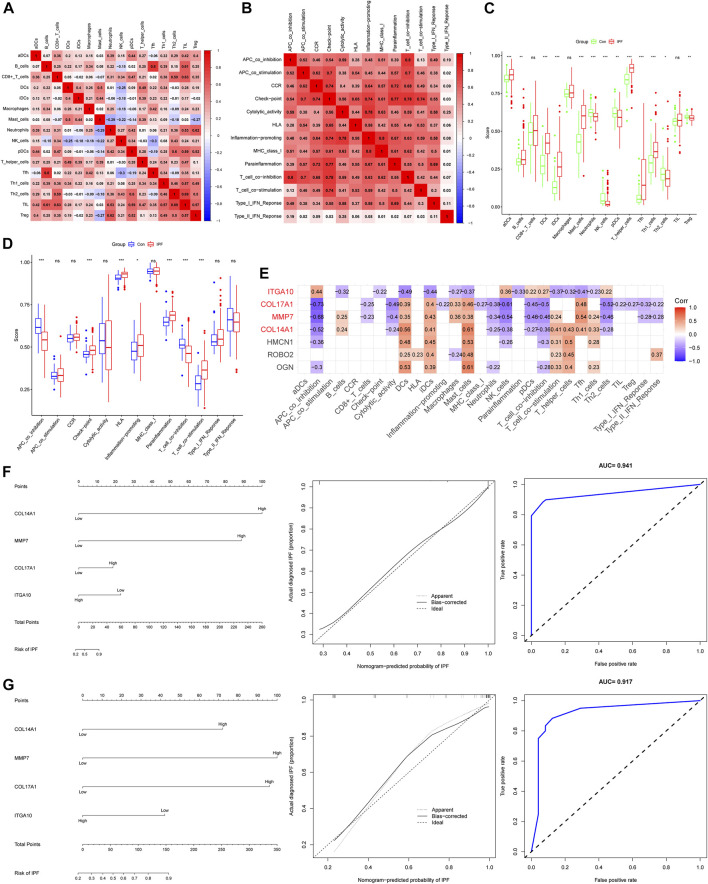
Immune infiltration analysis and establishment of IPF risk model. **(A)** Correlation heatmap of immune cells. **(B)** Immune function related heatmap. **(C)** Difference of immune cell expression between IPF and control groups. **(D)** Difference of immune function expression between IPF and control group. **(E)** Heatmap of correlation between IHGs expression and immune infiltration. **(F)** IHGs predicts the occurrence of IPF in training set. **(G)** IHGs predicts the occurrence of IPF the validation set.

We used the ssGSEA algorithm to evaluate immune infiltration in 265 samples (Supplementary Figure S2). In the correlation analysis of immune cells, the positive correlation between Tfh cells and B-cell was the strongest, and the correlation coefficient was 0.8. The negative correlation between Tfh cells and NK cells was the strongest, and the correlation coefficient was −0.3 (Figure 4A). Interestingly, we did not observe a negative correlation for immune function, whereas there was a positive correlation (r = 0.8) between T-cell co inhibition and APC co inhibition (Figure 4B). For immune cells, the expression of aDCs, B-cell, DCs, iDCs, Mast cells, T helper cells, Tfh and Th1 cells were increased in IPF, while the expression of neutrophils, NK cells, pDCs, Th2 cells and Treg cells were decreased (Figure 4C). For immune function, Check point, HLA, Inflammation promoting, T-cell co stimulation and Parainflamation were over-expressed in IPF, while T-cell co inhibition and APC co inhibition were underexpressed in IPF (Figure 4D). Finally, four IHGs were screened by correlation analysis, including *COL14A1*, *COL17A1*, *ITGA10* and *MMP7* (Figure 4E). These results suggest that the activation of multiple immune cells and the coordination of immune functions are important in the pathogenesis of IPF.

### 3.5 Construction and validation of IPF risk model

We created a logistic regression model of IHGs and used a nomogram to predict IPF risk (Figure 4F). The calibration curve used to evaluate the risk nomogram of IPF patients showed good consistency in this study. The results showed that the AUC of the training data set was 0.941, indicating that our model had good predictive ability. In order to further verify the prediction effect of our model, we used independent external validation dataset to verify (Figure 4G). The results showed that the calibration curve also showed good consistency in the training dataset. The AUC value in the validation dataset was 0.917, which also shows that our model had good predictive ability. Moreover, The C index also shows that our model had good predictive power. The C index was 0.941 (95% CI: 0.917–0.965) in the training dataset and 0.917 (95% CI: 0.840–0.994) in the validation dataset.

### 3.6 The expression level of IHGs in pan-cancer

**FIGURE 5:**
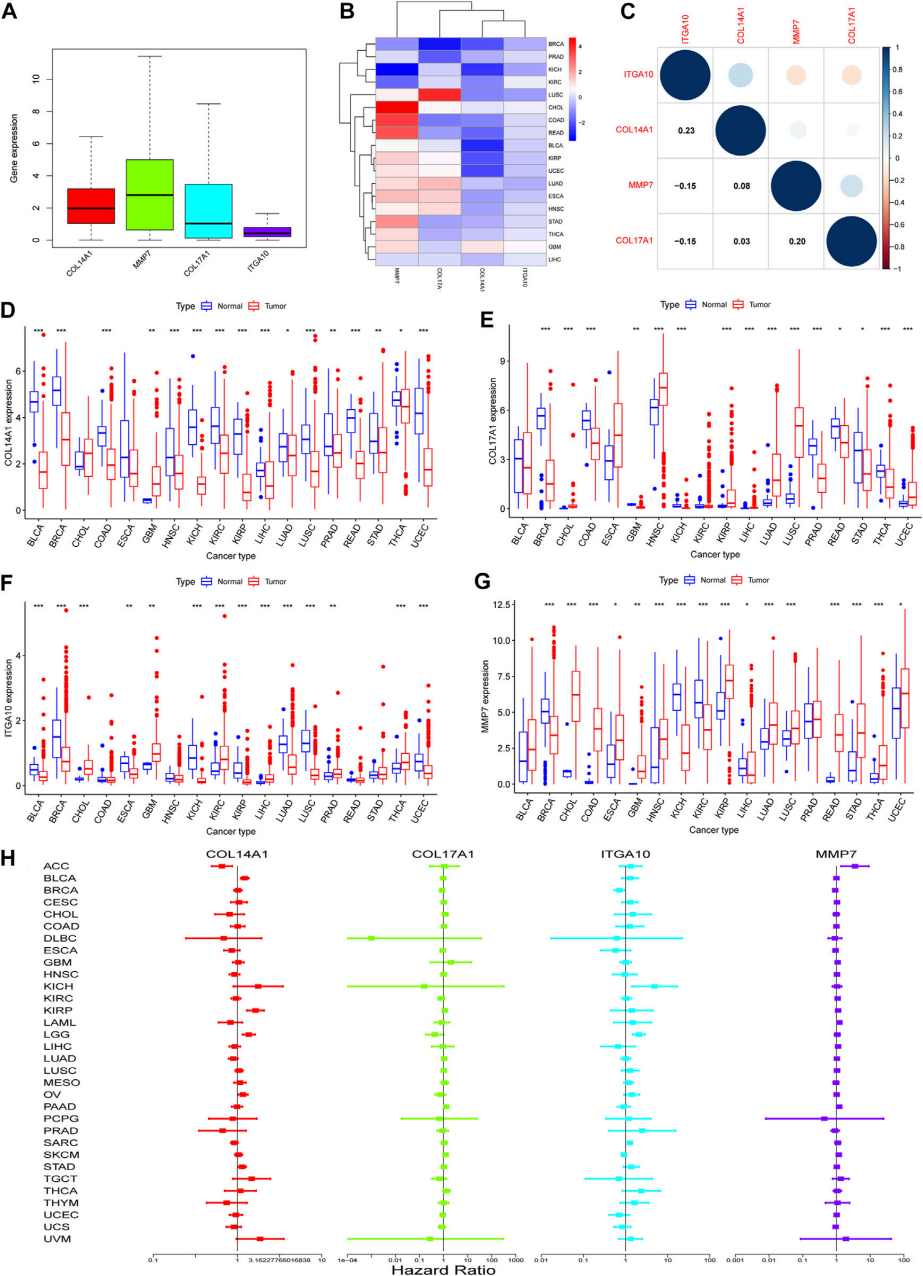
Expression of IHGs in Human Cancer. **(A)** Boxplots of IHGs expression levels in cancer. **(B)** Heatmap of IHGs expression levels in different cancer types and adjacent tissues. **(C)** Positive (blue) and negative (red) correlations between IHGs. Expression of *COL14A1* **(D)**, *COL17A1* **(E)**, *ITGA10* **(F)** and *MMP7* **(G)** in different tumor types and adjacent tissues. **(H)** Forest plot shows the relationship between IHGs expression and OS in 33 tumors.

To determine whether there are differences in the expression of IHGs in tumors, mRNA expression levels of IHGs in normal and tumor tissues were analyzed. As shown in Figure 5A, the expression of *MMP7* in IHGs was relatively high, while the expression of *ITGA10* was the lowest. The expression of IHGs in different cancer types is also quite different (Figure 5B). Overall, *COL14A1*, *COL17A1*, and *ITGA10* tended to be downregulated in most tumors, while *MMP7* tended to be upregulated in most tumors. Correlation analysis showed that *ITGA10* was weakly positively correlated with *COL14A1*, and weakly negatively correlated with *MMP7* and *COL17A1* (Figure 5C). Although there is a correlation between IHGs, the correlation coefficient value is between −0.15 and 0.23, which proves that the correlation is weak or negligible. *COL14A1* was highly expressed in 1 tumor and lowly expressed in 15 tumors (Figure 5D). *COL17A1* was significantly upregulated in seven tumors, while significantly downregulated in eight tumors (Figure 5E). *ITGA10* expression was increased in six tumors and decreased in eight tumors (Figure 5F). *MMP7* expression was higher in 12 tumors and lower in four tumors (Figure 5G).

### 3.7 Pan-cancer survival analysis of IHGs

**FIGURE 6:**
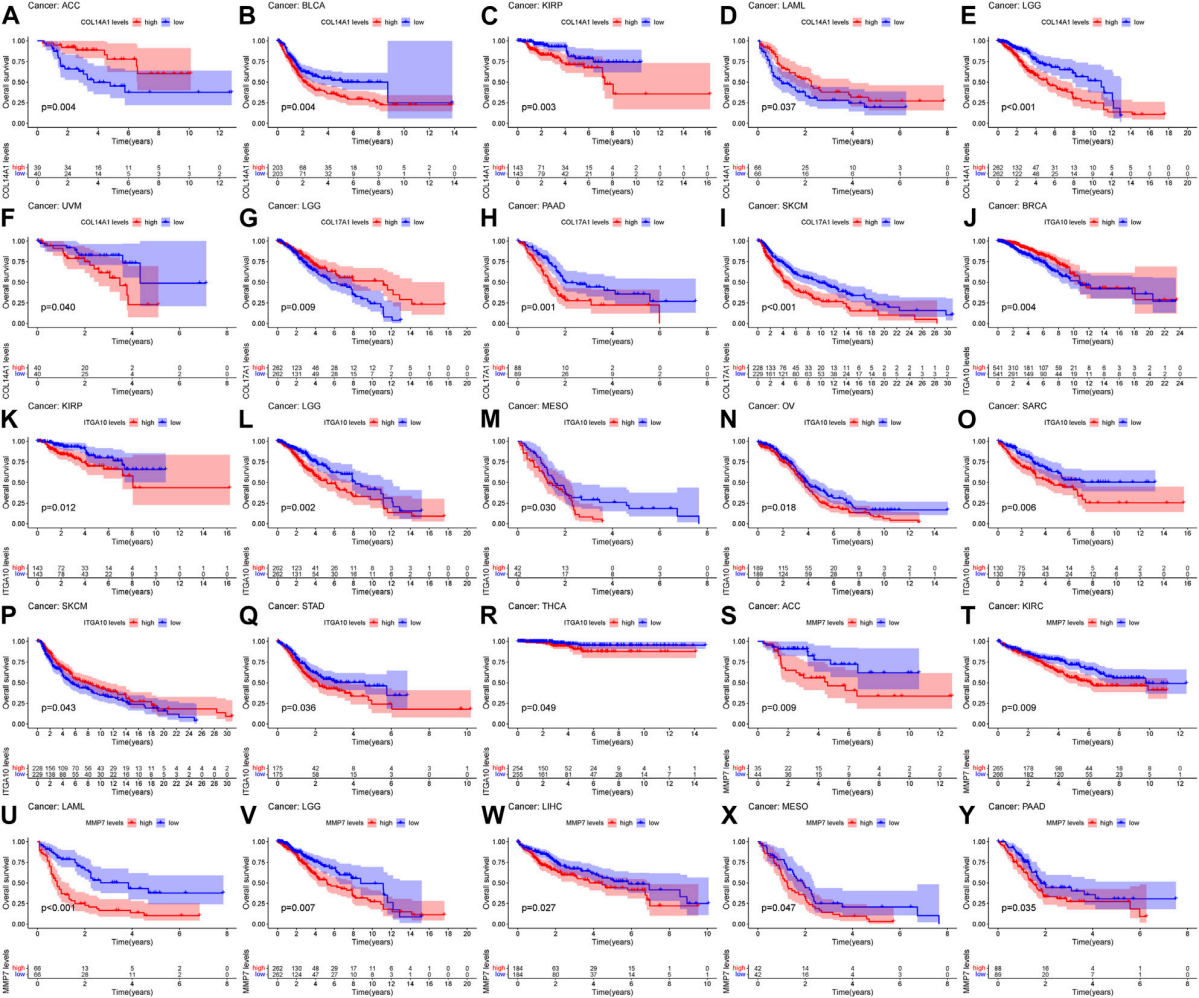
Relationship between IHGs expression and prognosis of different tumors. OS survival curves for *COL14A1* in six tumors: **(A)** ACC, **(B)** BLCA, **(C)** KIRP, **(D)** LAML, **(E)** LGG, **(F)** UVM. OS survival curves for *COL17A1* in 3 tumors:**(G)** LGG, **(H)** PAAD, **(I)** SKCM. OS survival curves for *ITGA10* in 9 tumors: **(J)** BRCA, **(K)** KIRP, **(L)** LGG, **(M)** MESO, **(N)** OV, **(O)** SARC, **(P)** SKCM, **(Q)** STAD,**(R)** THCA. OS survival curves for *MMP7* in 7 tumors: **(S)** ACC, **(T)** KIRC, **(U)** LAML, **(V)** LGG, **(W)** LIHC, **(X)** MESO, **(Y)** PAAD.

Based on the results of differential analysis, we used forest maps and survival curves to further understand whether IHGs expression was linked with tumor prognosis (Figure 5H). Cox regression analysis revealed that increased *COL14A1* expression was a negative factor affecting KIRP, LGG, BLCA, STAD and OV, while a positive factor affecting ACC. Increased expression of *COL14A1* was related to shorter overall survival (OS) in BLCA, KIRP, LGG and UVM, whereas decreased expression of *COL14A1* was related to shorter OS in ACC and LAML (Figures 6A–F). As shown as Figures 6G–I, Cox regression analysis revealed that *COL17A1* over-expression was an adverse factor for PAAD and SKCM, but a favourable factor for BRCA. Overexpression of *COL17A1* was linked with poorer OS in PAAD and SKCM, whereas increased *COL17A1* expression predicted favorable OS in LGG. As seen as Figures 6J–R, Cox regression analysis found that the increased expression of *ITGA10* was a negative factor for LGG, SARC and KICH, and a positive factor for SKCM and BRCA. High *ITGA10* expression was related to shorter OS for KIRP, LGG, MESO, OV, SARC, STAD, THCA, and longer OS for BRCA and SKCM. According to Figures 6S–Y, Cox regression analysis revealed that high expression of *MMP7* was an adverse factor for PAAD, ACC, LAML, KIRC, LIHC and SKCM, while high expression of *MMP7* was a beneficial factor for BRCA. Survival analysis showed that patients with increased *MMP7* expression in ACC, KIRC, LAML, LGG, LIHC, MESO, PAAD had shorter OS.

### 3.8 Correlation of IHGs expression with TME and tumor stemness

**FIGURE 7:**
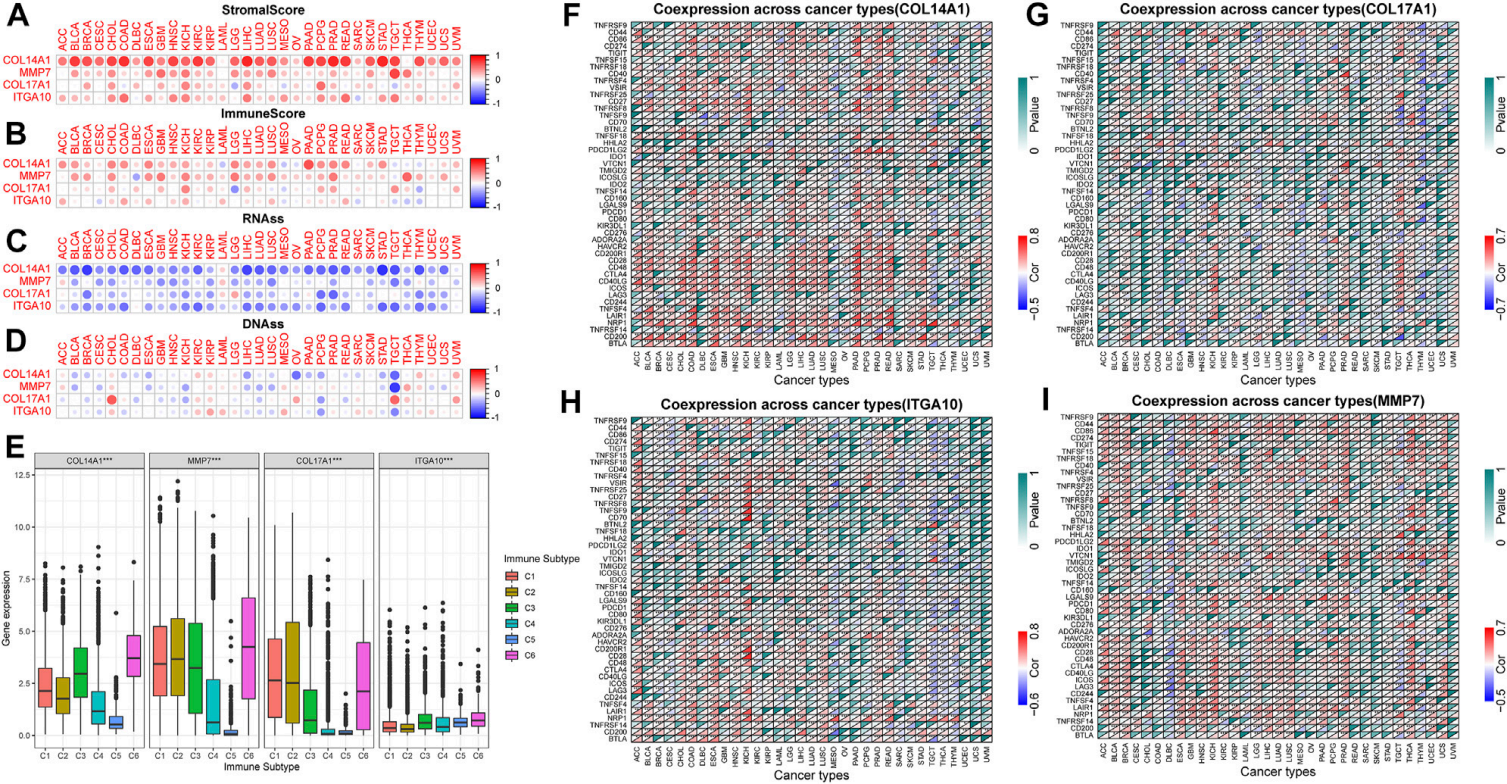
Correlation of IHGs expression with TME, stemness score, immune subtypes and immune checkpoints. **(A,B)** IHGs expression was related to immune and stromal scores in 33 tumors. **(C,D)** IHGs expression was related to stemness score in 33 cancers. **(E)** IHGs expression was associated with immune subtypes. The correlation between *COL14A1* **(F)**, *COL17A1* **(G)**, *ITGA10* **(H)** and *MMP7* **(I)** and immune checkpoints in 33 cancers.

TME is closely related to tumorigenesis and tumor cells escaping the immune system. The therapeutic effect and clinical prognosis of tumor are also correlated with TME. Therefore, the correlation between IHGs expression and tumor purity was assessed to understand whether IHGs are involved in tumor immunity. Overall, IHGs expression was positively related to stromal scores, with *COL14A1* having the strongest correlation with stromal score (Figure 7A). In terms of immune score, IHGs expression was positively correlated with CHOL, KICH, LIHC, and PCPG, etc., (Figure 7B). This suggests that IHGs have similar effects in the TME. Furthermore, we also evaluated the correlation between IHGs expression and tumor stemness score to understand the effect of IHGs expression on tumor differentiation. We found that IHGs were negatively related to RNAss in most tumors (Figure 7C). In contrast, it was positively related to DNAss in KIRC, KIRP, THYM and UVM (Figure 7D). These results suggest that the higher the expression of IHGs, the weaker the stemness score and the higher the degree of tumor differentiation.

### 3.9 Correlation of IHGs expression with immune subtype and immune checkpoints

Our results showed that *COL14A1*, *COL17A1*, and *MMP7* were over-expressed in the C1, C2, and C6 subtypes, and *ITGA10* was over-expressed in the C5 and C6 subtypes (Figure 7E). The high expression of *COL14A1*, *COL17A1* and *MMP7* was closely related to C1, C2 and C6 subtypes, indicating that these three genes may have carcinogenic effects. Moreover, we observed a significant correlation between IHGs expression and immune checkpoint genes in different tumor types. Specifically, the results revealed that *COL14A1* was positively related to immune checkpoints in most tumors except MESO, OV, SARC, TGCT, THCA, THYM, UCEC, UCS, and UVM (Figure 7F). *COL17A1* was significantly related to immune checkpoints in most tumors except ACC, CHOL, DLBC, UCS, and UVM (Figure 7G). *ITGA10* was positively related to more than 30 immune checkpoint genes in COAD, ESCA, KICH, HNSC and LUSC (Figure 7H). In addition to CESC, CHOL, COAD, SARC, SKCM, and UVM, *MMP7* was closely related to immune checkpoint genes (Figure 7I). These results indicate that IHGs expression is closely related to immune checkpoint genes, suggesting that IHGs may play a vital role in mediating tumor immune patterns.

### 3.10 Immune cell infiltration analysis of IHGs in pan-cancer

**FIGURE 8:**
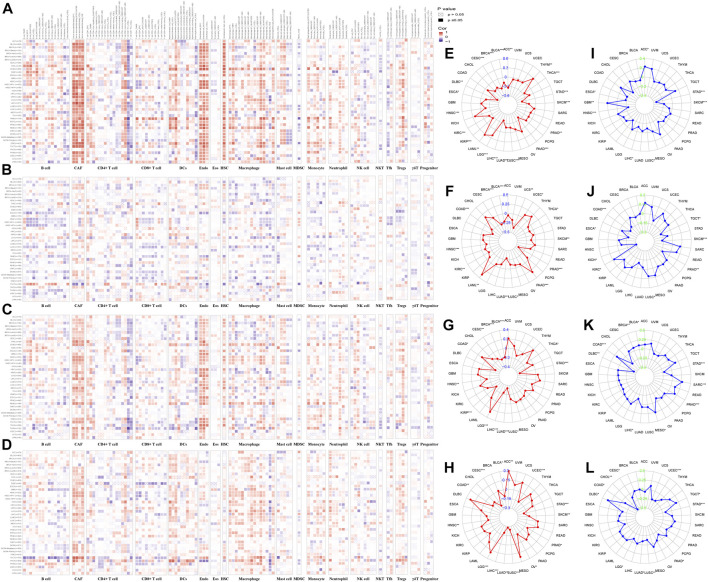
Correlation of IHGs expression with immune cell infiltration, TMB and MSI. The correlation between *COL14A1* **(A)**, *COL17A1* **(B)**, *ITGA10* **(C)** and *MMP7* **(D)** and 21 immune cells in 33 cancers. The correlation between *COL14A1* **(E)**, *COL17A1* **(F)**, *ITGA10* **(G)** and *MMP7* **(H)** and TMB in 33 cancers. The correlation between *COL14A1* **(I)**, *COL17A1* **(J)**, *ITGA10* **(K)** and *MMP7* **(L)** and MSI in 33 cancers.

We analyzed the correlation between tumor infiltrating immune cells and IHGs expression by TIMER2.0 to understand whether IHGs participated in tumor immune infiltration. We found that *COL14A1* was positively related to CAF, DCs, Endo, HSC, Macrophage, Monocyte and Tregs (Figure 8A). *COL17A1* was negatively related to most immune cells in HNSC, LUSC and ESCA (Figure 8B). *ITGA10* was positively related to CAF, Endo, HSC, neutrophils and Tregs in most tumors (Figure 8C). *MMP7* was positively related to CAF, DCs, Macrophage and Monocyte in most tumors (Figure 8D). Compared with *COL17A1*, *ITGA10* and *MMP7*, *COL14A1* had a higher correlation coefficient with infiltrating cells. These results suggest a potential mechanism by which IHGs have different prognostic value in different tumors.

### 3.11 TMB and MSI analysis of IHGs in human cancers

The more frequent the mutation of tumor cells, the more new antigens produced, making them more susceptible to immunotherapy (Gryfe et al., 2000; Samstein et al., 2019). Herein, we investigated the correlation between IHGs expression with TMB, MSI. We found that *COL14A1*/*ITGA10*/*MMP7* was positively related to TMB in LGG, and negatively related to TMB in STAD, LUSC, LUAD, LIHC, HNSC and BLCA (Figures 8E–H). As for MSI, *COL14A1*/*ITGA10*/*MMP7* expression was negatively correlated with STAD (Figures 8I–L). Although these correlations are important to guide immunotherapy in cancer patients, the correlation coefficients between IHGs expression and TMB and MSI did not exceed 0.6 in all tumor types, suggesting that IHGs are unlikely to affect tumorigenesis by participating in gene modification processes and are insufficient to independently predict patient response to immunotherapy.

### 3.12 Functional enrichiment analysis of IHGs in pan-cancer

**FIGURE 9:**
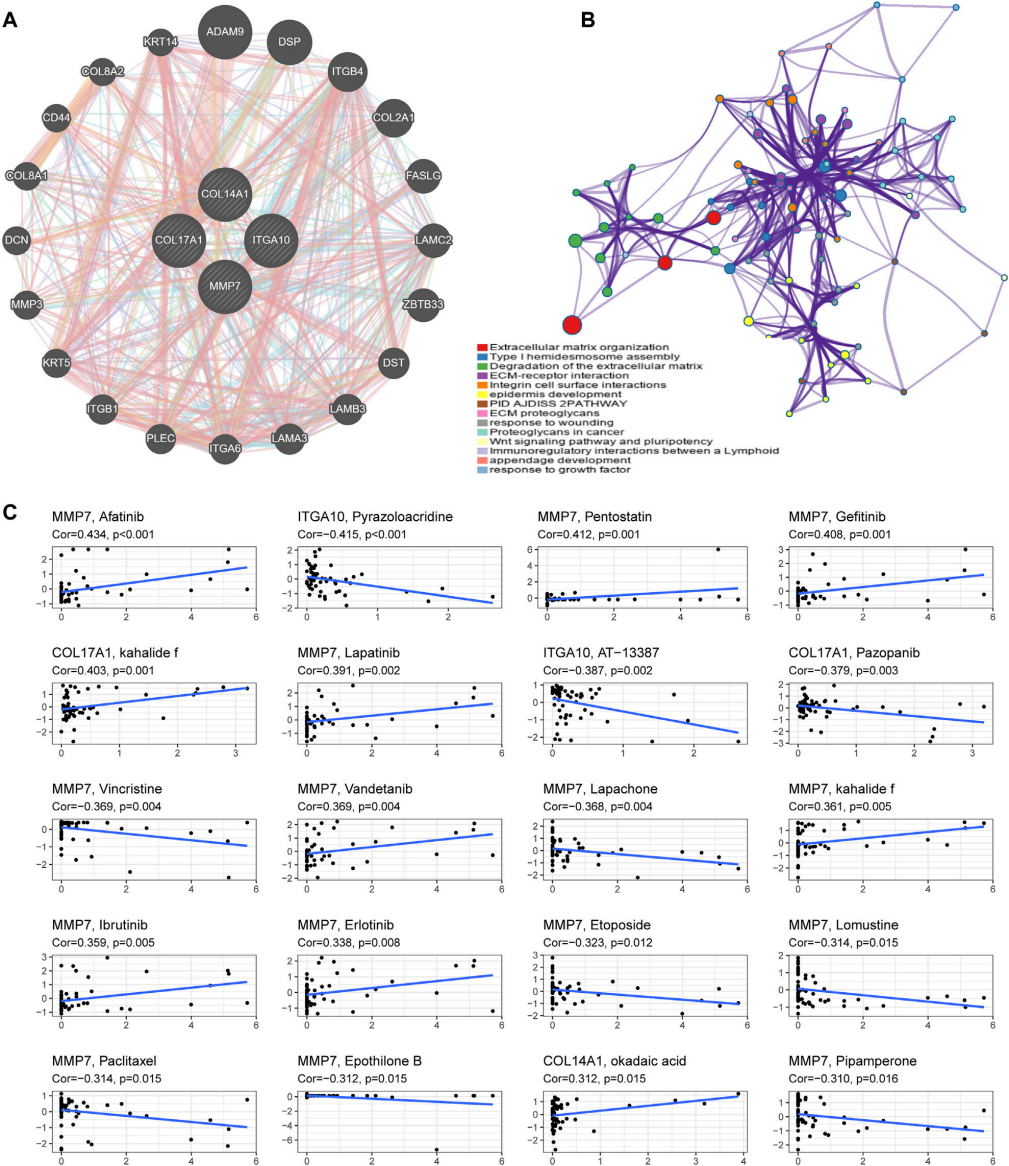
Functional enrichment analysis and drug sensitivity analysis of IHGs. **(A)** Construction of a PPI network with 24 genes centered on IHGs. **(B)** Enrichment analysis using Matescape. **(C)** Correlation analysis between IHGs and drug sensitivity of anticancer drugs in CellMiner.

We utilized GeneMANIA to screen genes associated with IHGs for comprehensive functional and pathway analysis of IHGs. Finally, we constructed an IHGs-centric PPI network consisting of 24 genes (Figure 9A). Metascape significantly enriched items include Extracellular matrix organization, ECM-receptor interaction, Degradation of the extracellular matrix, Integrin cell surface intercations, epidermis development, PID AJDISS 2PATHWAY, ECM proteoglycans, response to wounding, Proteoglycans in cancer, Wnt signaling pathway and pluripotency, Type I hemidesmosome assembly, Immunoregulatory interactions between a Lymphoid, appendage development and response to growth factor (Figure 9B).

### 3.13 Drug response analysis of IHGs

We further explored whether the expression of IHGs has guiding significance for clinical medication. Among 263 drugs (FDA approved or clinical trials), we found that the sensitivity of 55 drugs was significantly correlated with IHGs expression levels. As presented in Figure 9C, we show the top 20 drugs most significantly associated with IHGs. The upregulated expression of IHGs was associated with increased sensitivity to Afatinib, Pentostatin, Gefitinib, kahalide f, Lapatinib, Vandetanib, Ibrutinib, Erlotinib, and okadaic acid, and decreased sensitivity to Pyrazoloacridine, AT−13387, Pazopanib, Vincristine, Lapachone, Etoposide, Lomustine, Paclitaxel, Epothilone B, and Pipamperone. Overall, we found that IHGs expression was associated with treatment response, suggesting that IHGs may be involved in tumor drug resistance.

## 4 Discussion

In this study, we screened 13 BMDEGs using bioinformatics methods, among which 12 genes were over-expressed and 1 gene was lowly expressed. Subsequent GO enrichment analysis showed that all BMDEGs were mainly related to extracellular matrix tissue and collagen catabolic process, while KEGG enrichment analysis showed a certain correlation with Notch signaling pathway. Based on three machine learning algorithms, we screened seven disease candidate genes. Using external datasets, it was confirmed that *COL14A1*, *COL17A1*, *HMCN1*, *MMP7*, *OGN* and *ROBO2* were highly expressed in IPF, while *ITGA10* was lowly expressed in IPF. ROC curve analysis further confirmed that all disease candidate genes have diagnostic value in IPF, suggesting that they may have potential application prospects in the treatment of IPF. The findings suggest their potential usage for diagnostic value in IPF since they are highly expressed in IPF. The findings will eventually lead to future studies about the potential role of their involvement in IPF, and if their roles are confirmed, the potential application prospects in the treatment of IPF are likely to evaluated. Finally, four IHGs (*COL14A1*, *COL17A1*, *ITGA10*, *MMP7*) were screened out. We then construct a logistic regression model of IHGs and use a nomogram to predict IPF risk. The AUC of the training set was 0.941, and that of the verification set was 0.917. It shows that our model has good predictive ability, and these four genes are potential biomarkers of IPF. Our study provides a theoretical basis for studying the role of BMs-associated immune biomarkers in the pathogenesis of IPF, and provides promising research suggestions for subsequent studies.

In pan-cancer, the expression of IHGs was significantly different across cancers compared to comparable normal tissues. *COL14A1* was significantly downregulated in almost all cancer types. *COL17A1*, *ITGA10* and *MMP7* showed high intertumoral heterogeneity between tumor tissues and adjacent tissues. These results suggest that IHGs are a potential cancer biomarker.

Our study found that *COL14A1* was an adverse factor for KIRP, LGG, BLCA, STAD and OV, and a favourable factor for ACC. However, there is a lack of relevant studies to support the effect of *COL14A1* on the prognosis of these cancers, and more studies are needed to prove this. *COL14A1* encodes the alpha chain of type XIV collagen, which is linked with mature collagen fibers (Schuppan et al., 1990). It has been reported that *COL14A1* can affect arterial remodeling and participate in the occurrence of cardiovascular diseases (Weis-Müller et al., 2006; Guay et al., 2015). To our knowledge, there is a lack of literature on the role of *COL14A1* in IPF. When compared cancer with or without metastasis, it seems that further decrease of *COL14A1* has better outcome, but, this has to be tested in a larger scale to validate (Goto et al., 2015; Jiang et al., 2022). *COL14A1* methylation is an unfavorable prognostic factor for renal cell carcinoma, and low *COL14A1* expression seems to promote tumorigenesis of renal cell carcinoma (Morris et al., 2010). It seems that *COL14A1* is increased in IPF, while it is decreased in cancer. The differential expression of *COL14A1* may indicate the critical signaling that differentiates IPF - a disease with non-stopping fibroblast growth, from cancer-a disease with non-stopping malignant cell growth. Therefore, our studies suggest the critical and differentiating signaling may involve *COL14A1*, its function or signaling, and hopefully, that can be investigated by future studies.

*COL17A1* is one of the triple-helix collagen genes encoding collagen XVII, a type II transmembrane protein found in basal epithelial cells that can affect cell growth and migration (Natsuga et al., 2019; Kozawa et al., 2021). *COL17A1* also lacks relevant research in the field of IPF. Currently, research on *COL17A1* has focused on cancer and skin diseases (Nishie, 2020). Our study found that *COL17A1* over-expression was related to poor prognosis of SKCM and PAAD. Studies have shown that *COL17A1* is over-expressed in a variety of cancers (Thangavelu et al., 2016; Huang et al., 2022). In contrast, another study found that upregulation of *COL17A1* expression was related to better prognosis in breast cancer (Yodsurang et al., 2017). *COL17A1* inhibits cancer cell migration and invasion by inactivating AKT/mTOR pathway, and its over-expression is linked with longer survival in patients with invasive breast cancer (Lothong et al., 2021).

*ITGA10* is a type II collagen-binding integrin first isolated from chondrocytes (Camper et al., 1998). *ITGA10* has the highest content in cartilage tissue and plays a crucial part in the formation of growth plates during bone development (Bengtsson et al., 2005). From the current overall research situation, the research on *ITGA10* mainly focuses on cancer. Our study suggested that increased *ITGA10* expression was linked with poor prognosis of SARC and longer survival of BRCA and SKCM. Previous studies have reported that *ITGA10* promotes drug resistance and proliferation of osteosarcoma cells by the activation of PI3K/AKT signaling pathway (Li et al., 2021). Similarly, *ITGA10* promotes myxfibrosarcoma survival and metastasis by activating TRIO/RAC and RICTOR signaling pathways, and antitumor effects were observed in mouse xenografts after *ITGA10* inhibition (Okada et al., 2016). In addition, dysregulation and carcinogenic effects of *ITGA10* had been observed in lung cancer, prostate cancer, and thyroid cancer (Mertens-Walker et al., 2015; Su et al., 2015; Saftencu et al., 2019). These findings suggest that *ITGA10* acts as an oncogene. According to our knowledge, *ITGA10* has not been reported on IPF, and more attention should be paid to IPF.

Different from *COL14A1*, *COL17A1* and *ITGA10*, *MMP7* had been supported by numerous literatures in the research field of IPF. *MMP7* is the smallest member of the matrix metalloproteinase family. *MMP7* plays a crucial part in the pathogenesis of fibrosis by degrading extracellular matrix proteins and activating multiple signaling molecules (Niu et al., 2019; Mahalanobish et al., 2020). *MMP7* is a target gene of Wnt/β-catenin and highly expressed in proliferative epithelial cells of IPF (Zuo et al., 2002; Fujishima et al., 2010). Previous studies have identified *MMP7* as a potential biomarker for IPF. For instance, *MMP-7* was identified as a predictor of survival in a combined model incorporating clinical parameters and *MUC5B* genotype (Peljto et al., 2013; Biondini et al., 2021). Consistent with previous studies, we found increased *MMP7* expression in IPF and negatively correlated with decreased FVC (Bauer et al., 2017). Some studies have shown that *MMP7* combined with other biomarkers may improve the survival prediction of IPF patients (Song et al., 2013; Hamai et al., 2016). In addition, a phase II clinical study showed that *MMP7* protein levels decreased in a dose-dependent manner after using JNK inhibitors, indicating that the use of *MMP7* to track IPF progression has potential clinical benefits (van der Velden et al., 2016). However, a recent study found that there was no difference in the baseline concentration of *MMP7* between IPF patients with or without disease progression, and short-term changes in its concentration could not reflect disease progression (Raghu et al., 2018; Khan et al., 2022). Another study indicated that *MMP7* was over-expressed in patients with subclinical interstitial lung disease and under-expressed in patients with mature IPF compared to healthy controls (Drakopanagiotakis et al., 2018). It is suggested that *MMP7* can be used as a potential marker for early detection of IPF. Besides, more and more evidences support *MMP7* as an oncogene involved in tumor cell proliferation, migration and apoptosis (Sun et al., 2021; Van Doren, 2022). *MMP7* is highly expressed in many cancers, and its expression is related to survival time and tumor stage (Lee et al., 2006; Liao et al., 2021). Knockdown of *MMP7* gene can inhibit tumor proliferation, migration and reduce drug resistance (Sanli et al., 2013; Yuan et al., 2020). Therefore, *MMP7* is expected to become a potential biomarker for evaluating tumor prognosis and a new target for tumor therapy.

GO enrichment analysis showed that BMDEGs were involved in the composition of basement membrane, extracellular matrix and endoplasmic reticulum cavity, and were related to extracellular matrix tissue, collagen metabolism and metallopeptidase activity. Alveolar epithelial damage and abnormal tissue repair are considered to be key factors in the development of IPF, which ultimately leads to the recruitment and activation of myofibroblasts to produce collagen-rich extracellular matrix. The deposition of extracellular matrix in IPF mainly involves matrix metalloproteinases (MMPs) and tissue inhibitors of metalloproteinases (TIMPs). More and more studies support the key role of MMPs in the pathogenesis of pulmonary fibrosis. Interestingly, some MMPs have pro-fibrotic effects, while others seem to play a protective role. In our study, we found that *MMP7* was over-expressed in IPF patients, and previous studies have shown that it contributes to the progression and adverse consequences of IPF, while *MMP19* seems to have a protective effect (Jara et al., 2015). Changes in the extracellular matrix of IPF can also affect the transcription of lung fibroblasts, resulting in abnormal translation of ECM proteins (Zolak and de Andrade, 2012). In addition, the degradation product of ECM (matrikines) also acts as a signal molecule and plays a central role in the fibrosis of IPF. Existing evidence suggests that ECM plays an important role in driving the circulation of pathogenic disease signals mediated by integrins, growth factors, matrikines, and MMPs (Hewlett et al., 2018). It is worth noting that the increased stiffness of ECM tissue is also a key driver of the fibrosis process. Compared with healthy lung scaffolds, collagen, proteoglycan and ECM glycoprotein in IPF scaffolds increased, but specific BMs proteins (such as laminin and collagen IV) decreased (Elowsson Rendin et al., 2019). New treatments for these ECM-driven processes are expected to bring benefits to IPF patients. Moreover, KEGG enrichment analysis showed that BMDEGs were related to Notch signaling pathway. It is reported that Notch signaling pathway plays a key role in the development, balance and regeneration of the respiratory system (Kiyokawa and Morimoto, 2020). The disorder of Notch signaling pathway is related to the occurrence of IPF, and the activation of Notch signaling can accelerate pulmonary fibrosis (Yang et al., 2022). Therefore, regulating the activation of Notch signaling pathway may be a new anti-fibrosis treatment strategy.

Although the pathogenesis of IPF remains unclear, a growing number of studies have implicated immune activation in its pathogenesis. Therefore, we used ssGSEA to further dissect the immune infiltration of the disease. In our results, we found that the expression of IHGs was correlated with different degrees of immune cell infiltration. The role of neutrophils in IPF remains unclear. On the one hand, inhibition of neutrophil chemokine *CXCL8* or lack of neutrophil elastase can reduce bleomycin-induced pulmonary fibrosis (Gregory et al., 2015; Gschwandtner et al., 2017). Similarly, increased neutrophils are associated with decreased FVC and all-cause mortality in IPF patients (Achaiah et al., 2022). It has been reported that deletion of exon 18 of *COL17A1* in mice leads to IL-17-related inflammatory responses in the skin and infiltration of eosinophils, neutrophils, T-cell and mast cells (Lindgren et al., 2023). XIV collagen is a neutrophil chemotactic factor that plays a role in neutrophil recruitment in rat inflammation (Nakagawa et al., 1999). On the other hand, the increase of neutrophils in BALF was not significantly related to the survival rate of IPF patients (Tabuena et al., 2005). Fibrosis caused mice infected with bacteria to show a higher mortality rate through the destruction of neutrophils (Warheit-Niemi et al., 2022). However, whether neutrophils have prognostic value in IPF is unclear, and further studies are needed to confirm the role of neutrophils in IPF. B-cell were increased in patients with IPF, which is consistent with our current findings. Activation of immune responses and increased infiltration of B-cell and macrophages are associated with IPF development (Xu et al., 2021). Previous studies have shown that B-cell and BLyS are elevated in patients with IPF and are inversely associated with patient outcome (Heukels et al., 2019). Consistent with increased B-cell activation, plasma IgA was elevated in IPF patients and inversely correlated with FVC (Heukels et al., 2019). These findings suggest that inhibition of B-cell activation has potential therapeutic value for IPF. In addition, our study showed a decrease in Tregs in IPF patients, which is consistent with previous findings. There is conflicting evidence supporting the role of Tregs in IPF. Tregs were originally thought to have anti-fibrotic effects. Tregs in peripheral blood and BALF were significantly reduced in IPF patients and were associated with decreased FVC (Kotsianidis et al., 2009). Subsequent studies have shown that tregs can promote fibrosis. The decrease of Tregs in peripheral blood and BALF of IPF patients is related to the degree of fibrosis (Peng et al., 2014) In the bleomycin-induced PF model, depletion of Tregs resulted in reduction of fibrosis, while induction or metastasis of Tregs resulted in worsening of fibrosis (Birjandi et al., 2016). Tregs may play different roles in different stages of fibrosis. Tregs play a pro-fibrotic role in the early stage of PF and a protective role in the late stage (Boveda-Ruiz et al., 2013). Tregs can promote collagen deposition and release of TGF-β in the early stage of PF (Lo Re et al., 2011). Tregs depletion attenuates PF by promoting Th17 response and regulating the shift of disturbed Th1/Th2 balance to Th1 dominance in lung tissue (Xiong et al., 2015). Consequently, we speculate that Tregs regulate different T-cell subsets at different stages of pulmonary fibrosis, which may explain the different roles of Tregs in pulmonary fibrosis. Existing studies have shown that inhibition of macrophage migration inhibitory factor (MIF) can downregulate the expression level of *ITGA10* and reduce bleomycin-induced pulmonary inflammation and fibrosis in rats (Luo et al., 2021). The relationship between *ITGA10* and MIF is still lacking relevant evidence and the future development of inhibitors targeting MIF may contribute to the treatment of pulmonary fibrosis. It has been reported that activated *MMP7* is located on alveolar macrophages and proliferative epithelial cells (Fujishima et al., 2010). Future studies are needed to further confirm how IHGs participate in the pathological process of IPF by affecting immunity.

We further explored the correlation between IHGs expression and TME, immune subtypes and immune cell infiltration. Our study found that IHGs expression was linked with different levels of immune and stromal cell infiltration. Further analysis revealed that *COL14A1* was positively correlated with CAF, DCs, Endo, HSC, Macrophage, Monocyte and Tregs. *ITGA10* was positively correlated with CAF, Endo, HSC, Neutrophil and Tregs. *MMP7* was positively correlated with CAF, DCs, Macrophage and Monocyte. The correlation between *COL17A1* and immune cells was not prominent. In addition, we also found that *COL14A1*, *COL17A1*, and *MMP7* were associated with more invasive immune subtypes, including C1, C2, and C6 subtypes. These results suggest that changes in immune and stromal cell composition make IHGs have different clinical features and immunotherapy responses. The relationship between IHGs expression and TME needs to be further studied at cellular and molecular levels.

We also explored the relationship between IHGs expression and immune checkpoint, tumor stemness score, TMB, and MSI. The results showed that IHGs expression was significantly related to RNAss and DNAss in most tumors. Previous studies have reported that higher stemness scores are linked with stronger tumor stem cell dedifferentiation and active biological processes, suggesting potential targets for chemotherapy drug development in cancer patients (Malta et al., 2018). IHGs expression level and immune checkpoint analysis showed that there was significant correlation between IHGs expression level and immune checkpoint in different tumors. Previous studies have shown that TMB is a good biomarker for predicting immunotherapy response in tumor patients (Goodman et al., 2017; Chan et al., 2019). MSI is also associated with prognosis, and high MSI indicates better prognosis (Ganesh et al., 2019). Our study suggested that IHGs expression was significantly related to TMB and MSI. These results reveal that immunotherapy may have potential benefits for cancer patients and may also help clinicians quickly identify patients who respond to immunotherapy. However, more studies at the molecular level are needed to fully explain the relationship between IHGs and immune response.

However, our research also has some shortcomings. Firstly, the biological mechanisms of *COL14A1*, *COL17A1*, *ITGA10* and *MMP7* in IPF and cancer are still unclear. Second, the results of this study need to be confirmed by relevant animals and human experiments. In the future research, we will continue to pay attention to the role of *COL14A1*, *COL17A1*, *ITGA10* and *MMP7* in IPF.

## 5 Conclusion

In summary, our study shows that BMs and immune disorders are closely associated with IPF. The IPF risk model based on IHGs showed that the high expression of *COL14A1*, *COL17A1*, *ITGA10* and *MMP7* was positively related to the risk of IPF. It was further confirmed that the AUC of the training set was 0.941 and that of the verification set was 0.917, indicating that *COL14A1*, *COL17A1*, *ITGA10* and *MMP7* were potential biomarkers for predicting the risk of IPF. Pan-cancer analysis showed that IHGs were related to prognosis, immune infiltration and drug sensitivity of cancer patients, and were expected to become new biomarkers for cancer patients. However, multicenter, large-scale and prospective studies are needed to confirm our results before *COL14A1*, *COL17A1*, *ITGA10* and *MMP7* are applied clinically.

## Data Availability

Publicly available datasets were analyzed in this study. This data can be found here: The row data included in this study are available in GEO (https://www.ncbi.nlm.nih.gov/geo/, (accessed on 16 August 2022)) and UCSC Xena (https://xena.ucsc.edu/, (accessed on 24 June 2022)).
