# Supplementary material for: Identification of immune biomarkers associated with basement membranes in idiopathic pulmonary fibrosis and their pan-cancer analysis
Source: Front Genet. 2023 Mar 2;14:1114601. doi: 10.3389/fgene.2023.1114601 (PMC10017543; doi:10.3389/fgene.2023.1114601)
Supplement: Supplementary file 1 [file DataSheet1.docx]

Supplementary Material

**Table S1**. AUC value of candidate disease genes in training set.

| Gene | AUC | 95%CI |
| --- | --- | --- |
| COL14A1 | 0.964 | 0.942-0.981 |
| COL17A1 | 0.915 | 0.881-0.947 |
| HMCN1 | 0.961 | 0.939-0.979 |
| ITGA10 | 0.946 | 0.902-0.980 |
| MMP7 | 0.937 | 0.907-0.962 |
| OGN | 0.894 | 0.850-0.934 |
| ROBO2 | 0.856 | 0.810-0.905 |

**Table S2**. AUC value of candidate disease genes in validation set.

| Gene | AUC | 95%CI |
| --- | --- | --- |
| COL14A1 | 0.881 | 0.761-0.968 |
| COL17A1 | 0.949 | 0.897-0.988 |
| HMCN1 | 0.813 | 0.706-0.906 |
| ITGA10 | 0.707 | 0.570-0.838 |
| MMP7 | 0.910 | 0.847-0.962 |
| OGN | 0.719 | 0.585-0.842 |
| ROBO2 | 0.600 | 0.459-0.732 |


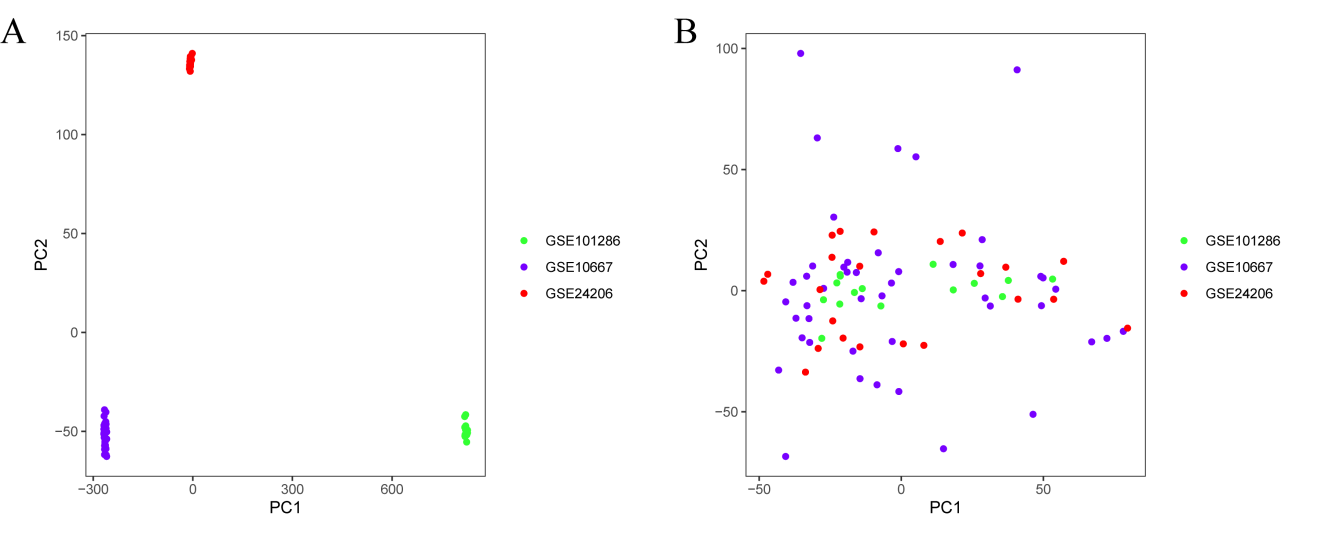


**Figure S1**: The PCA plot of the validation group dataset. (A)PCA diagram before eliminating batch effect. (B)PCA diagram after eliminating batch effect.


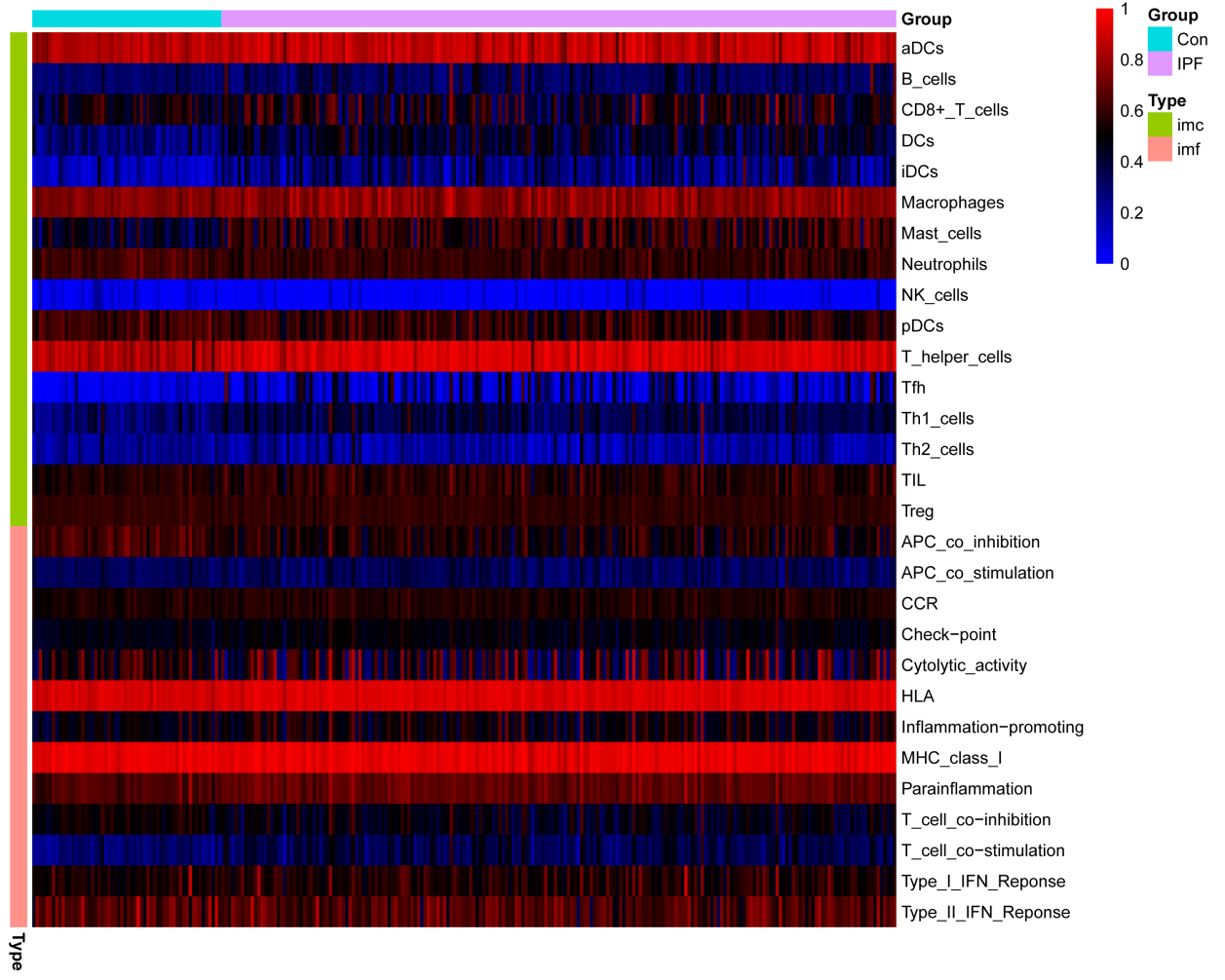


**Figure S2**: Heatmap of immune cell enrichment and immune function in IPF and control groups.
